# Application of the Chinese Aircraft-shaped Sleeve system in the treatment of tibial shaft fractures using a suprapatellar approach for tibial intramedullary nailing: a randomised controlled trial

**DOI:** 10.1186/s13018-018-0995-7

**Published:** 2018-11-14

**Authors:** Ke Lu, Ting-ting Zhou, Yi-jun Gao, Hong-zhen Wang, Zhi-qiang Wu, Yuan Wang, Dong-gui Zheng, Qi-rong Dong

**Affiliations:** 1 Department of Orthopaedics, the Second Affiliated Hospital of Soochow University, 1055 Sanxiang Road, Suzhou, 215004 Jiangsu China; 2 Department of Joint Surgery, the First People’s Hospital of Kunshan, Suzhou, 215300 Jiangsu China; 3 Department of Radiology, the First People’s Hospital of Kunshan, Suzhou, 215300 Jiangsu China; 4 R&D Department, Jiangsu OKANI Medical Technology Co., Lt, Suzhou, 215214 Jiangsu China

**Keywords:** Suprapatellar approach, Tibial fracture, Intramedullary nailing, Sleeve, Magnetic resonance, Patellofemoral joint

## Abstract

**Background:**

The use of the suprapatellar approach for intramedullary nailing has recently become popular for the treatment of tibial fractures. This study aimed to investigate the effectiveness of using the Chinese Aircraft-shaped Sleeve (CASS) system on the clinical outcomes of tibial intermedullary nailing using a suprapatellar approach for the treatment of tibial fractures in a cohort of adult Chinese patients.

**Methods:**

Magnetic resonance images (MRI) of the knee joints of 212 healthy adults with normal lower limb function from May 2011 to May 2015 were obtained from a level I Chinese regional trauma centre. Sixty inpatients at the same trauma centre who underwent treatment for tibial shaft fractures from June 2015 to June 2018 were enrolled. Tibial intramedullary nailing fixation of tibial shaft fractures via the suprapatellar approach was performed with either a CASS system or a conventional sleeve. The measurements of patellofemoral joint anatomy, the surgical time, the assessments of the patellofemoral joint cartilage conditions, and information of residual debris based on arthroscopic analysis were subsequently collected.

**Results:**

The mean patellar angle (PA), sulcus angle (SA), sulcus width (SW) and sulcus depth (SD) were 135.40 ± 6.20°, 142.37 ± 5.33°, 33.37 ± 2.73 mm, and 4.29 ± 0.63 mm, respectively. The surgical time until entry reaming commencement and the irrigation time were significantly lower in the CASS group (*P* < 0.001). The difference in cartilage damage rate between groups was statistically significant (*P* = 0.031); the difference in residual debris conditions was not statistically significant (*P* = 0.1967).

**Conclusion:**

The use of the CASS system could improve clinical outcomes of intramedullary nailing via suprapatellar approach for patients with a small patellofemoral joint space.

## Background

Tibial fractures are one of the most common long bone fractures in the human body. Intramedullary nailing (IMN) has become the preferred treatment choice for tibial fractures, particularly those of the shaft, as it is minimally invasive and ensures high fixation strength, which allows patients to return to routine activities immediately after surgery [1]. The traditional surgical approach for IMN begins with an incision below the patella (in the middle or along the patellar ligament), with an insertion of the nail with the knee joint flexed to at least 90°. In 1996, Tornetta and Collins [2] first reported a suprapatellar approach, performed with the knee in a semi-extended position (in about 15° of flexion), using a mid-line incision, from the superior patellar pole to the tibial tubercle, followed by the creation of a slit through two-thirds of the medial patellar retinaculum to sublux the patella.

**Fig. 1:**
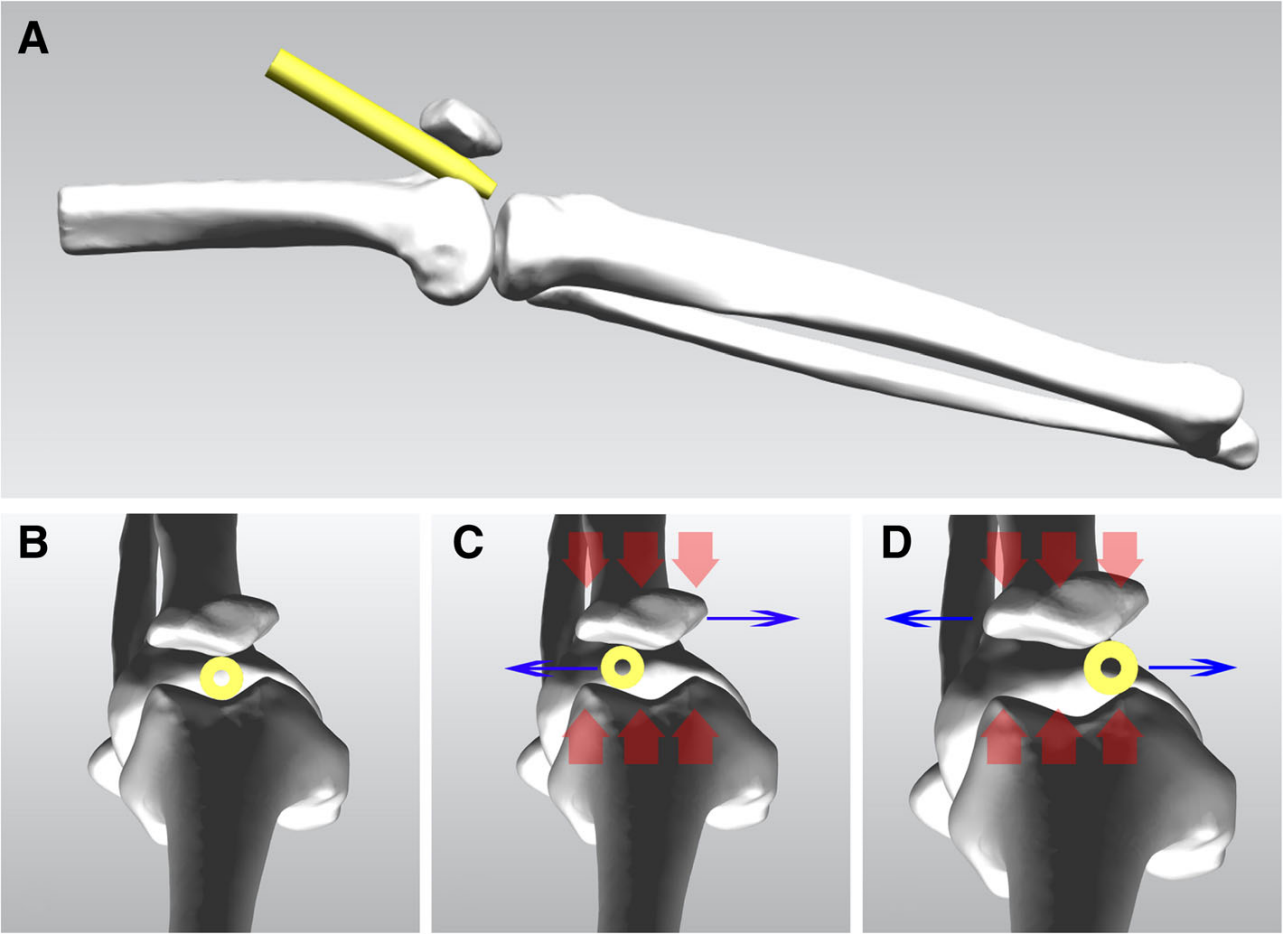
Instability in the position of the conventional sleeve during intramedullary nailing. **a**, **b** The conventional sleeve must be located in the central area of the patellofemoral joint, as viewed from the lateral and axial positions. **c**, **d** The conventional sleeve is round, such that when the pressure on the patellofemoral joint (red arrow) is high, with the patella slides to one side (blue arrow) and the sleeve to the opposite side (blue arrow)

Although advances have been made to improve the suprapatellar approach, several technical issues remain. Foremost, existing tools are designed for Western populations and, therefore, may not be appropriate for all other populations. As an example, individuals of Chinese ethnicity tend to be smaller than Westerners and, as such, the patellofemoral joint space is relatively smaller, resulting in greater resistance after incision of the quadriceps and insertion of the outer protective sleeve, compared to the original procedure developed in Westerners. The small patellofemoral joint space also increases the risk of iatrogenic injury to the intra-articular structure, as well as making placement of the guide wire more difficult, which increases operative time. Second, the original protective sleeve used in the original suprapatellar IMN approach is V-shaped, which is a poor match for Chinese individuals. Due to this shape mismatch, when the pressure on the patellofemoral joint is high, the patella slides to one side and the sleeve to the opposite side (Fig. 1), with this instability increasing the difficulty of accurately insert the IM nail. Finally, a large amount of debris is generated during surgical access and reaming, requiring a thorough irrigation prior to incision closure. Ensuring debris evacuation from the joint cavity has become a difficult problem that needs to be addressed. In view of these difficult issues associated with the performance of a suprapatellar approach of IMN among patients with a relatively small patellofemoral joint space, we present in this study a newly designed protective sleeve system that is specifically based on the anatomical parameters of patellofemoral joints in Chinese individuals and describe appropriate modifications to the conventional surgical procedures. Our aim, specifically, was to investigate the effectiveness of the sleeve system in the treatment of tibial shaft fractures using a suprapatellar approach for tibial IMN.

## Methods

### Anatomical parameters of the Chinese Aircraft-shaped Sleeve (CASS) system

The study was approved by our institutional review board and ethics committee and all participants provided informed consent.

Magnetic resonance images (MRI) of the knee joints of 212 healthy adults with normal lower limb function were obtained from May 2011 to May 2015 from a level I Chinese regional trauma centre. The exclusion criteria were as follows: previous history of knee surgery, trauma, space-occupying lesions in the knee joint and surrounding areas, patellar softening, sulcus dysplasia, synovial hyperplasia, and severe fluid accumulation in the joints. Of the 212 patients, 101 were men and 111 were women, and the age range was 18–73 years (mean 41 years). In total, there were 106 left knees and 106 right knees. The MRIs were obtained using a 1.5-T system (Philips 1.5-T Achieva, Philips, Netherlands) with patients in the supine position with full knee extension. Axial T2-weighted (TR/TE: shortest/100, matrix 256 × 192, FOV 160 × 160, slice thickness 3 mm) sequences were obtained. MRIs were analysed on a workstation (Centricity RIS CE V3.0, General electric medical system (China) Co., Ltd., USA) by one radiologist, and measurements were made using electronic callipers.

**Fig. 2:**
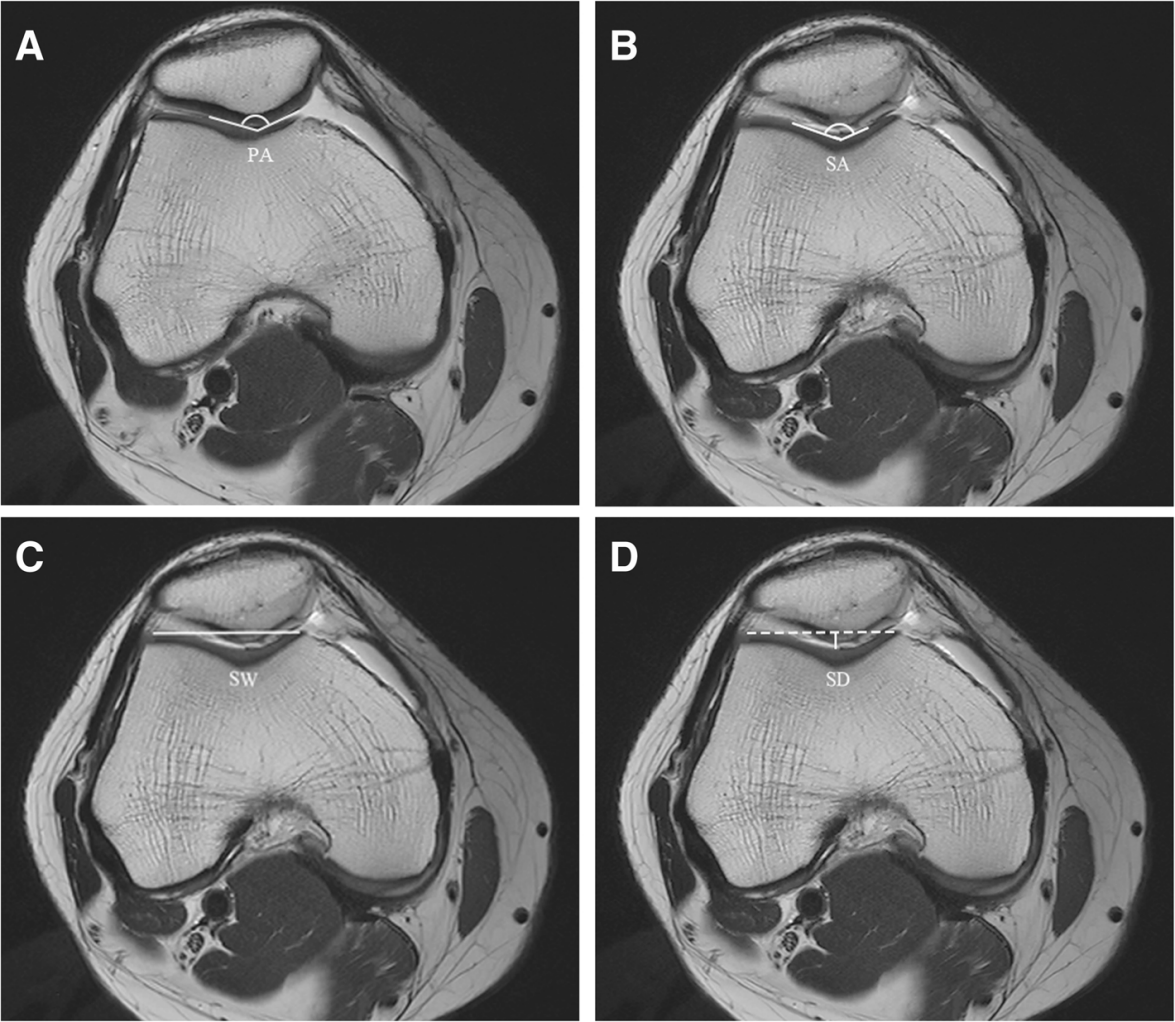
The following anatomical parameters of the patellofemoral joint (PFJ) anatomy were measured on magnetic resonance images: **a** Patella angle (PA): for the angle where the medial and lateral cartilage surfaces of the patella meet. **b** Sulcus angle (SA): the angle between the medial and lateral cartilage surfaces of the sulcus. **c** Sulcus width (SW): the length of the line connecting the most prominent points of the medial and lateral cartilages inside the trochlear groove. **d** Sulcus depth (SD): the length of the perpendicular line from the deepest point between the cartilage of the trochlear groove cartilage and the SW

The patellar angle (PA) was measured on the axial image of the knee where the patella was the widest. The sulcus angle (SA), sulcus width (SW), and sulcus depth (SD) were measured on axial images of the knee where the transepicondylar axis was fully displayed on the first level from the proximal to the distal ends (Fig. 2).

### Application of the CASS system

Sixty inpatients who underwent treatment for a tibial shaft fracture at our centre, between June 2015 and June 2018, were enrolled. The selection criteria were as follows: (1) tibial shaft fractures (OTA type 42), (2) achievement of mature bone, and (3) the use of the Expert Tibial Nail system (ETN; DePuy Synthes, USA), with a suprapatellar approach. The exclusion criteria were as follows: (1) knee injury or stiffness, (2) patella baja, (3) patellar ligament calcification, (4) existing vascular nerve injury, (5) soft tissue injury around the incision area, and (6) metabolic or pathological bone diseases. After patients were enrolled in the study, each received a sealed envelope containing a randomised number, generated using SAS 9.2 (SAS Institute, Inc., Cary, NC, USA) based on the order of admission, for allocation to either the CASS group or the conventional sleeve group, in a 1:1 ratio.

The general characteristics of the patient group were as follows: 33 men and 27 women, with an age range of 22 to 69 years. There were no significant differences in baseline conditions between the CASS and conventional sleeve group. All procedures were performed by one senior surgeon. Due to the specific nature of the intervention methods, double-blinding could not be achieved between the surgeon and patients. Data processing, statistical analysis, and evaluations were performed by independent professionals who had no knowledge of group assignment.

### Sample size

Based on preliminary tests and a previous study [3], we initially defined the primary outcome as the rate of cartilage damage, with an estimated rate of 0.03 in the treatment group (CASS group) and 0.28 in the control group (conventional sleeve group). The ratio between the sample sizes of the two groups was 1:1. Using an *α* value of 0.05 and *β* value of 0.2 (power of 0.80), the minimum requirement, based on the SAS sample size calculator procedure [4], was 30 patients per group.

### Surgical treatment

**Fig. 3:**
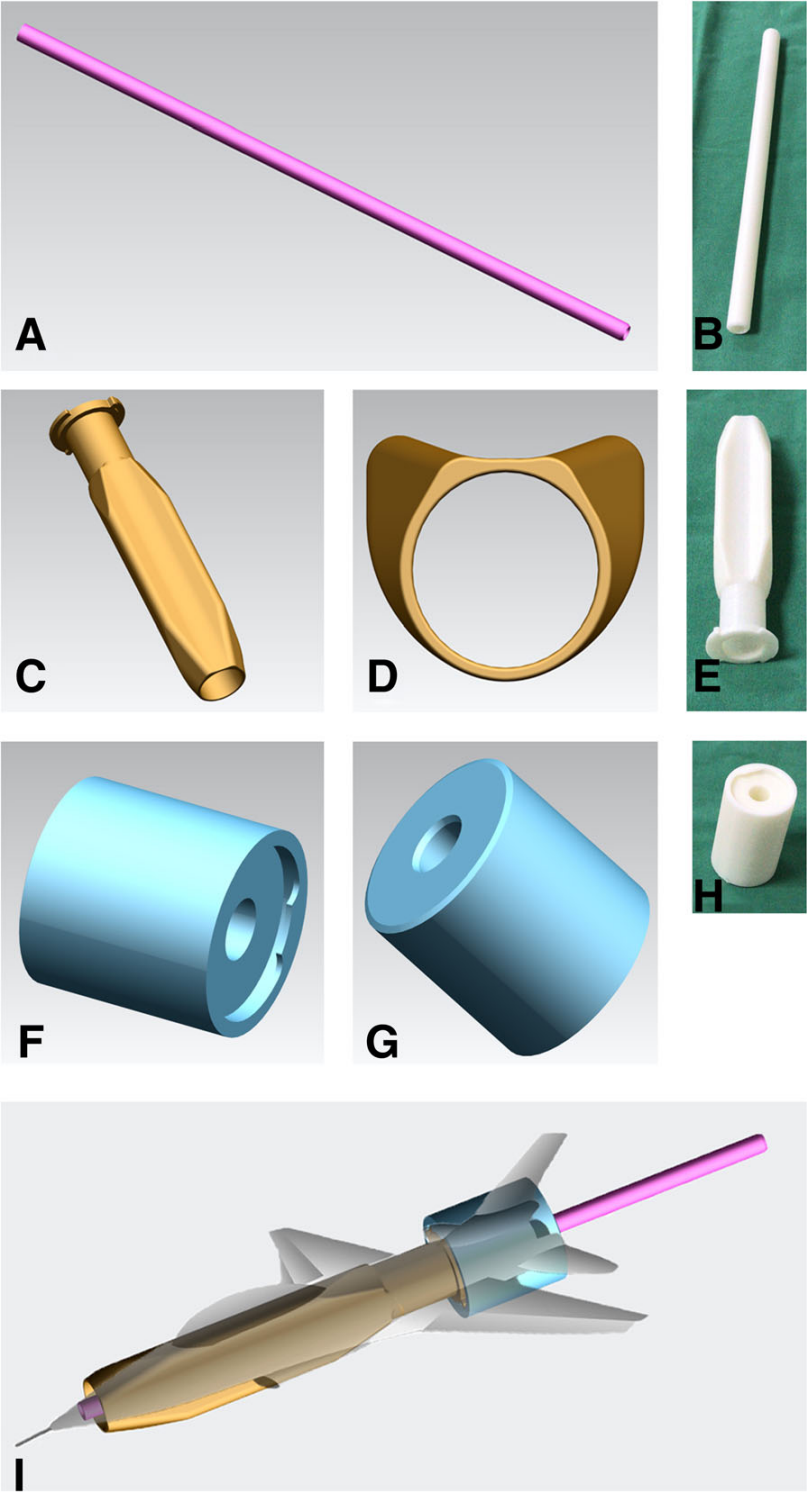
The Chinese Aircraft-shaped Sleeve system: **a**, **b** Positioning of the protective sleeve for the guide-wire-first method (aircraft cabin). **c**–**e** Outer protective sleeve (aircraft wings). **f**–**h** Connector (aircraft tail). **i** When the three components are combined into one image, that shape of aircraft sleeve is obtained

**Fig. 4:**
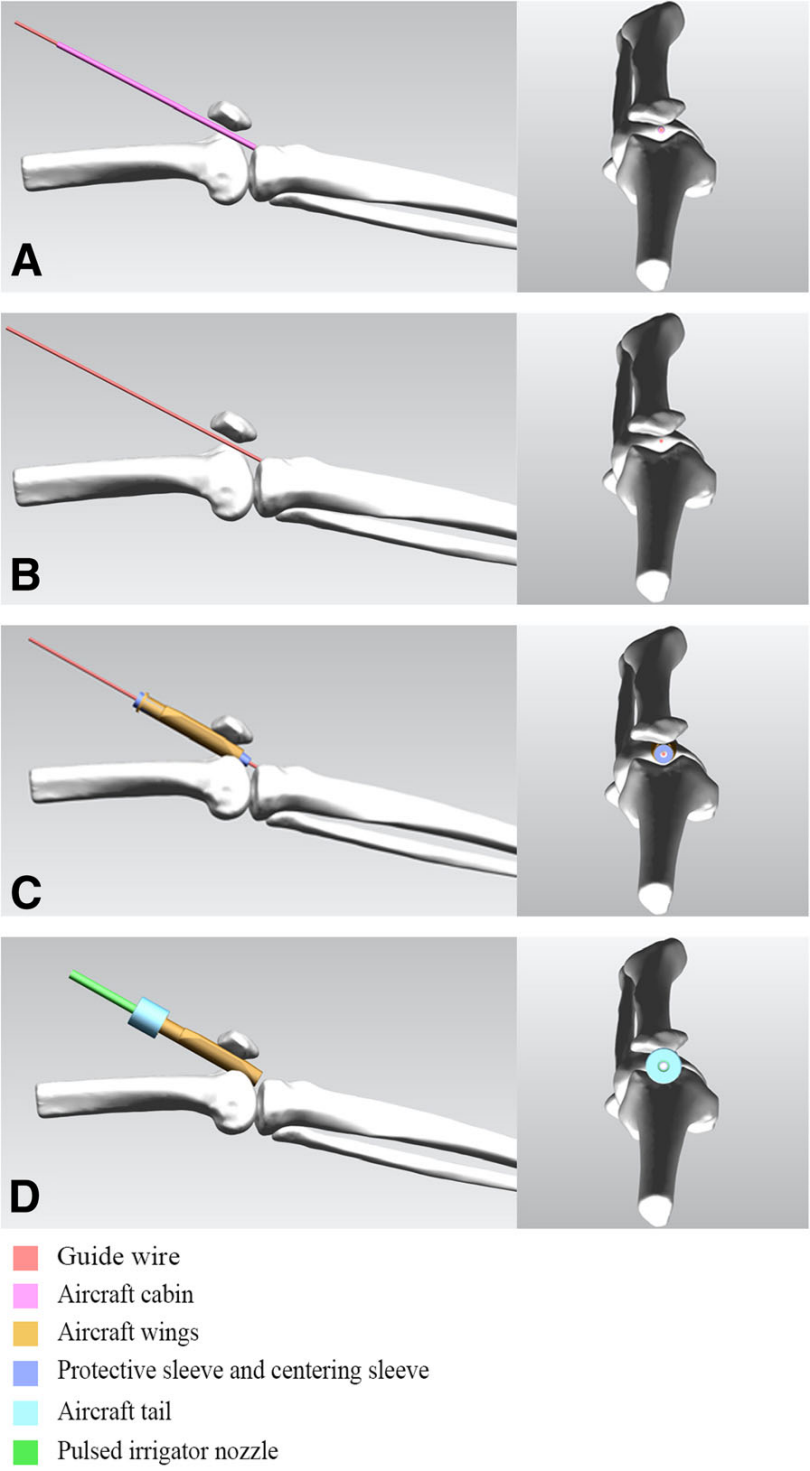
Surgical procedure using the Chinese Aircraft-shaped Sleeve system: **a** The aircraft cabin protects the guide wire as it is inserted into the optimal position in the patellofemoral joint. **b** Removal of the aircraft cabin. **c** The outer aircraft wings, the conventional metal inner protective sleeve, and the centring sleeve are inserted along the guide wire, and the centring sleeve is subsequently pulled out to facilitate routine procedures, such as the opening of the medullary canal. **d** Installation of the aircraft tail on the end of the aircraft wings prior to irrigation, and insertion of a pulsed irrigation nozzle for thorough rinsing of the joint cavity

**Fig. 5:**
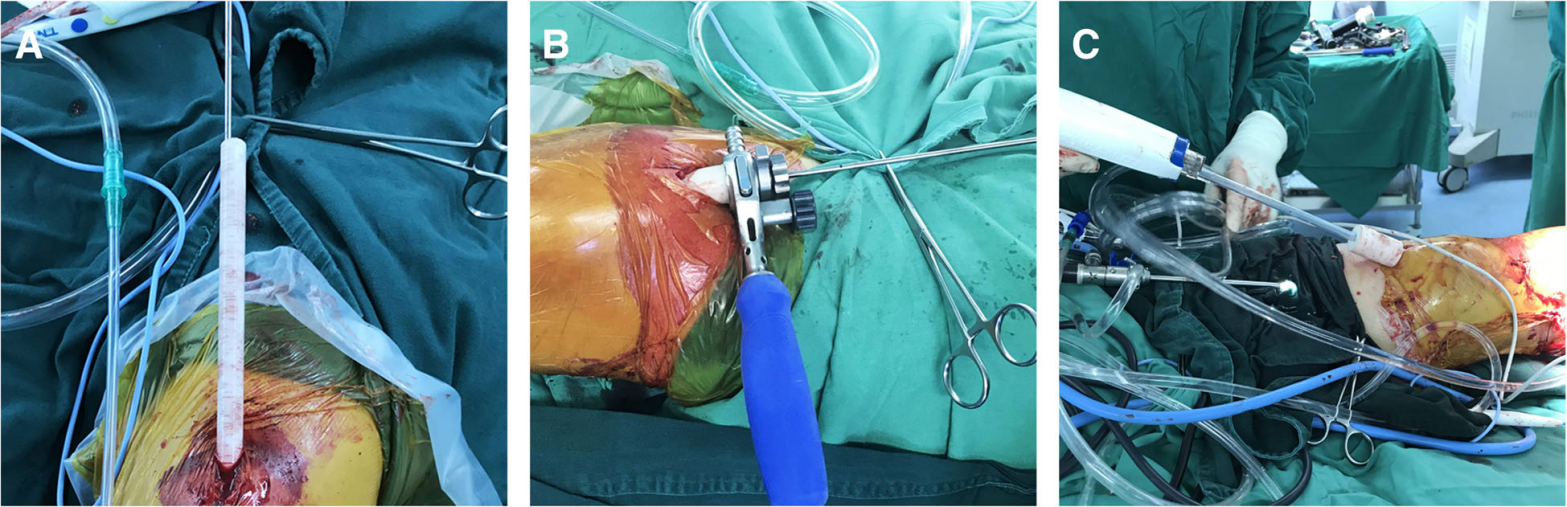
Chinese Aircraft-shaped Sleeve system surgical technique: **a** Advanced positioning technology combining the aircraft cabin and the guide wire. **b** Reinforcement and stabilisation of the aircraft wings. **c** Rinsing technique, combining the aircraft tail and pulsed irrigation

Patients in the CASS group underwent IMN using the original suprapatellar ETN system but with the CASS, which was designed based on the anatomical parameters of the patellofemoral joints measured from the MRI of the sample of 212 healthy individuals, and 3D printed (Fig. 3). The CASS was fabricated using thermoplastic polyurethane (TU), which has a stiffness of 42D–64D. The surgical procedure includes the following (Figs. 4 and 5): preoperative positioning of the patient in the supine position, with a knee pillow under the popliteal fossa of the affected limb to position the knee joint in a semi-extended position (flexion of 10°-20°). A longitudinal skin incision, of approximately 2–4 cm, was made, 1.5 cm above the superior patellar pole. The subcutaneous soft tissue was also incised. Deep separation was performed, with the quadriceps tendon being cut and the joint capsule above the patella opened further. An index finger was used to reach the joint capsule for preliminary separation of soft tissue. The patella was revealed anteriorly. The protective sleeve device was then placed, before placement of the guide wire. To address the shape mismatch in the CASS group, the positioning sleeved (aircraft cabin) was custom-designed based on the average knee parameters for Chinese individuals, using a translucent material. After opening the joint capsule, the positioning sleeve was gently inserted into the femoral sulcus, up to the tibial guide wire insertion point, and a 3.2-mm guide wire was drilled into it. An intraoperative radiograph was obtained to ensure optimal positioning of the insertion and position of the guide wire. The use of the compact, translucent positioning sleeve facilitated manoeuvring of the guide wire within the patellofemoral joint for accurate positioning. The positioning sleeve was removed, and the sleeve system was inserted (including the handle, inner protective sleeve, and centring sleeve) along the guide wire. The inner layer of the sleeve body has a hollow cylindrical shape for inserting a conventional metal circular inner sleeve. The outer layer is O-shaped in the axial position and extends on both sides to become V-shaped, thus resembling aircraft wings, which further stabilise the sleeve, such that even if high pressure is applied to the patellofemoral joint, the sleeve will not slide relative to the patella. Similar to the conventional infrapatellar approach, the entry hole of the nail was made in the direction of the guide wire after removal of the centring sleeve. Closed or open reduction was then performed. A guide wire was inserted for reaming followed by the appropriate intramedullary nail. The distal and proximal locking screws were fixed, and the end cap was inserted. After pulling out the metal guiding device, the surgical space was irrigated thoroughly. We also designed a connector (aircraft tail) for the end of the sleeve to improve flushing. The head has a cross-lock design that can be tightly joined or quickly separated by a rotation. The cross section of the tail is a hollow circle, similar in size to the pulsed irrigation nozzle (WJ-06, Wanjie Medical Device Co., Ltd., China). This section allows passage of the irrigation nozzle, with the nozzle reaching the area surrounding the opening of the tibia through the hollow tube, allowing for thorough irrigation and aspiration of the debris from the joint cavity. In this study, we used 4000 ml of sterile saline for irrigation. The incision was then closed in layers.

The conventional sleeve group underwent surgery using traditional suprapatellar procedures for the ETN system (DePuy Synthes, Raynham, MA, USA). The incision and joint cavity were irrigated repeatedly with a solution volume of 4000 ml.

### Clinical outcomes

The following outcomes were obtained for analysis: surgical time, calculated up to the point of remaining; arthroscopic assessment of the condition of the patellofemoral joint cartilage at two time points (once after deep separation, before sleeve insertion, and once after insertion of the end cap), using a Smith & Nephew arthroscope system (Smith & Nephew, Andover, MA, USA); the Outerbridge scale [5] which provides a grading of cartilage damage (grade 0, normal; grade I, cartilage with swelling and softening; grade II, fragmentation and fissuring in an area ≤ 0.5 in. in diameter; grade III, fragmentation and fissuring in an area > 0.5 in. diameter; and grade IV, erosion of the cartilage down to the bone); and the irrigation time. The number of cases with (positive) or without (negative) cartilage damage and residual debris was recorded for both groups. Arthroscopic evaluations were performed by a sports medicine expert, who did not have knowledge of group allocation.

### Statistics

Statistical analysis was performed using SAS 9.2 (SAS Institute, Inc., Cary, NC, USA). The data collected for PA, SA, SW, and SD were divided into two groups by sex. A *t* test was used to compare the sample means, adopting a completely randomised design. The Shapiro-Wilk test was used to test data normality, and the *F* test was used to evaluate the homogeneity of variance. The chi-square test, with correction for continuity in a 2 × 2 table, was used to compare the cartilage damage rate and the residual rate of debris between the groups. *P* < 0.05 indicated statistical significance.

## Results

The mean ± standard deviation of SA was 142.37 ± 5.33°, with no statistically significant difference between sexes (*P* = 0.513). The mean ± standard deviation for PA was 135.40 ± 6.20°, SW was 33.37 ± 2.73 mm, and SD was 4.29 ± 0.63 mm, with a significant between-sex difference (*P* < 0.001; Table 1).

**Table 1:** Measurements of patellofemoral joint anatomy in 212 normal adult Chinese people

Measured parameter	Men (*n* = 101)	Women (*n* = 111)	Total	*P* value*
Patella angle (°)	137.30 ± 6.02	133.68 ± 5.87	135.40 ± 6.20	< 0.001
Sulcus angle (°)	142.62 ± 5.32	142.14 ± 5.36	142.37 ± 5.33	0.5128
Sulcus width (mm)	35.17 ± 2.23	31.72 ± 2.01	33.37 ± 2.73	< 0.001
Sulcus depth (mm)	4.54 ± 0.58	4.06 ± 0.58	4.29 ± 0.63	< 0.001

*Student’s *t* test

**Fig. 6:**
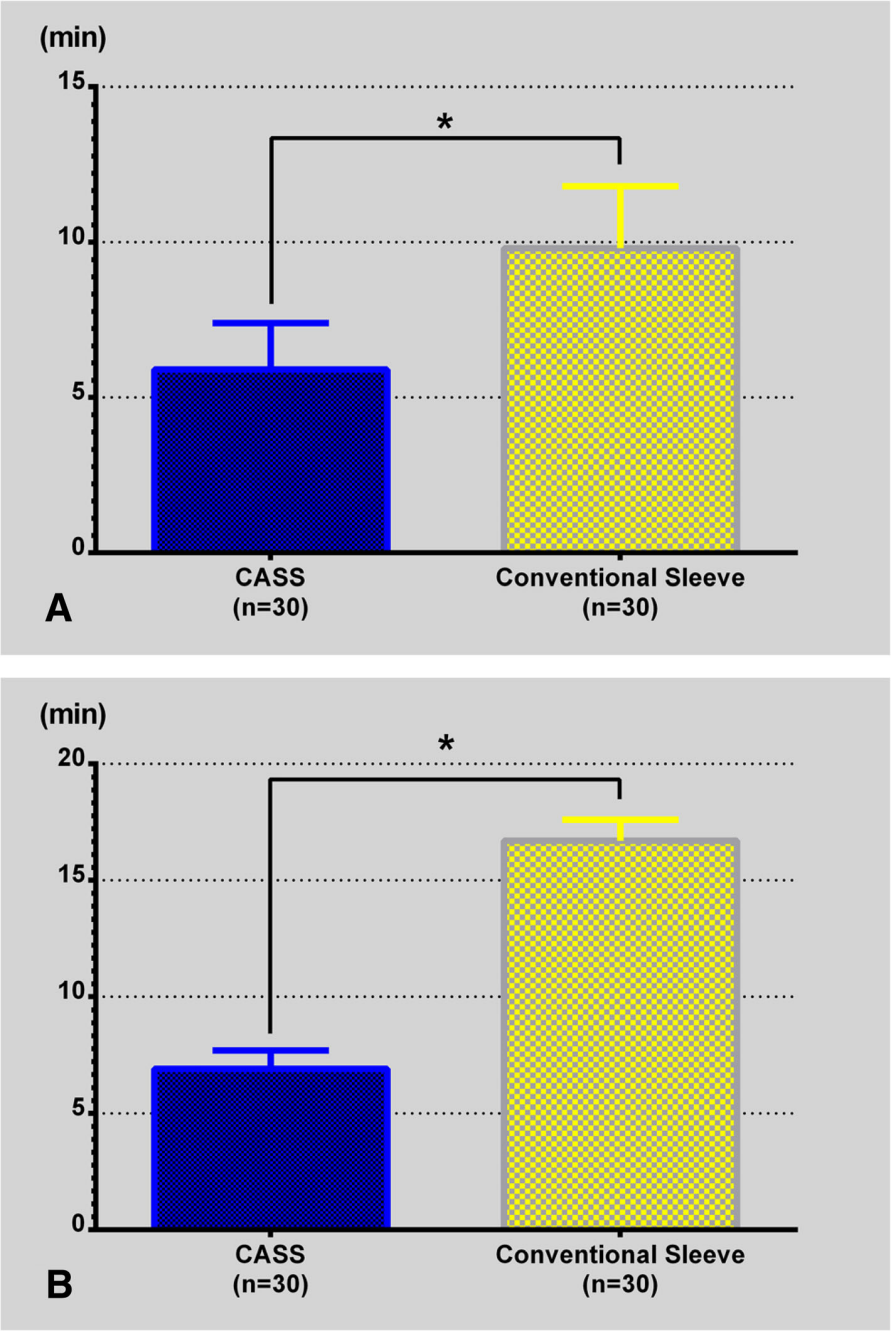
Comparison of surgical times: **a** Surgical time until entry reaming commencement. **b** Irrigation time. Overall, use of the CASS system shortens the surgical time. CASS: Chinese Aircraft-shaped Sleeve system, *P < 0.0001

In the CASS group, the surgical time until entry reaming commenced in the CASS group was 5.9 ± 1.5 min, compared to 9.8 ± 2.0 min in the conventional sleeve group, which was a significant difference (*P* < 0.001; Fig. 6a). The CASS group irrigation time was 6.9 ± 0.8 min, whereas it was 16.7 ± 0.9 min for the conventional sleeve group. The use of the aircraft tail and the pulsed irrigation device saved an average of 9.8 min of operative time, and the difference was significant (*P* < 0.001; Fig. 6b).

**Fig. 7:**
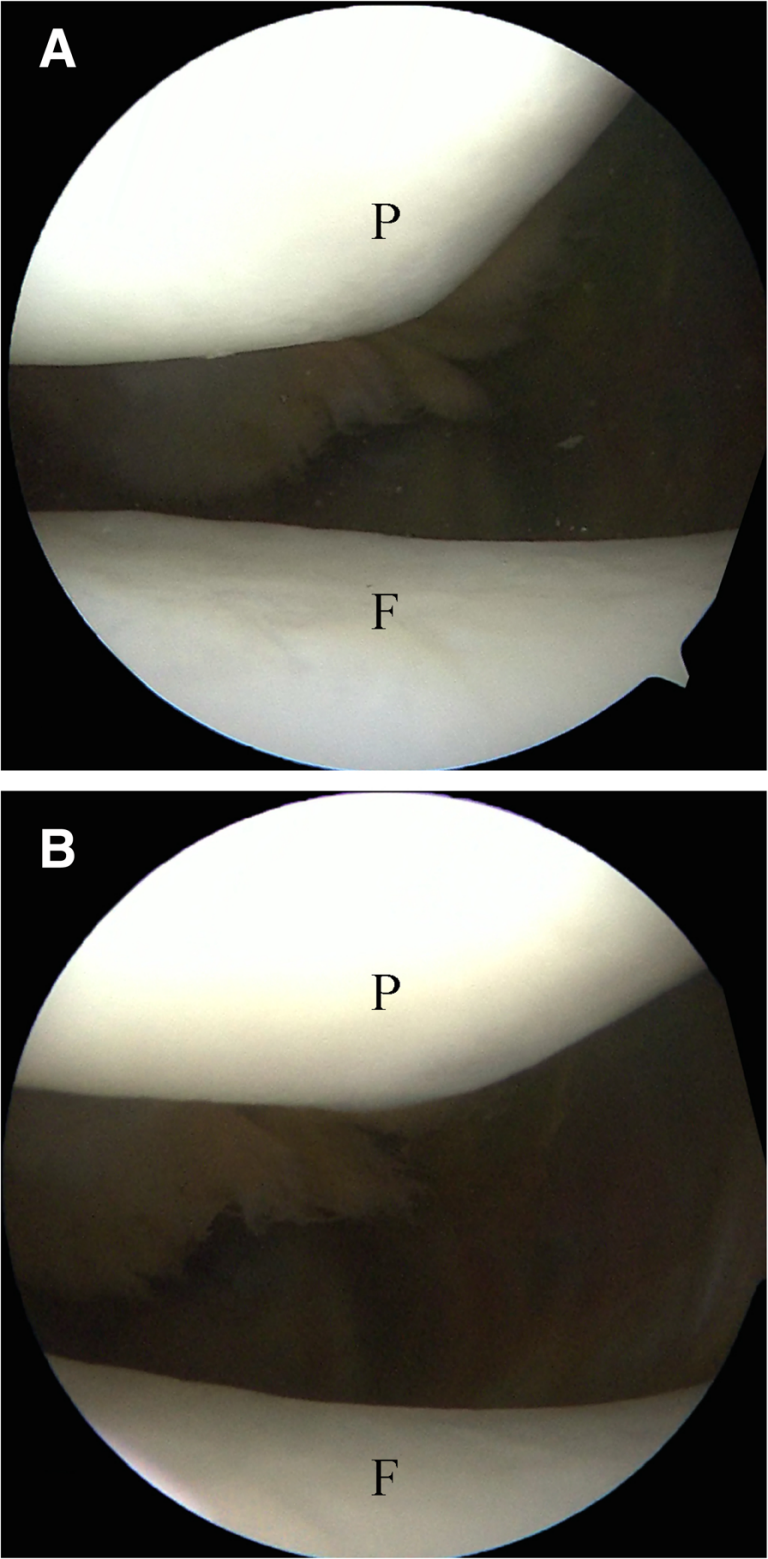
Arthroscopic assessment: **a** Preoperative Outerbridge scale: normal. **b** Postoperative Outerbridge scale: normal

**Fig. 8:**
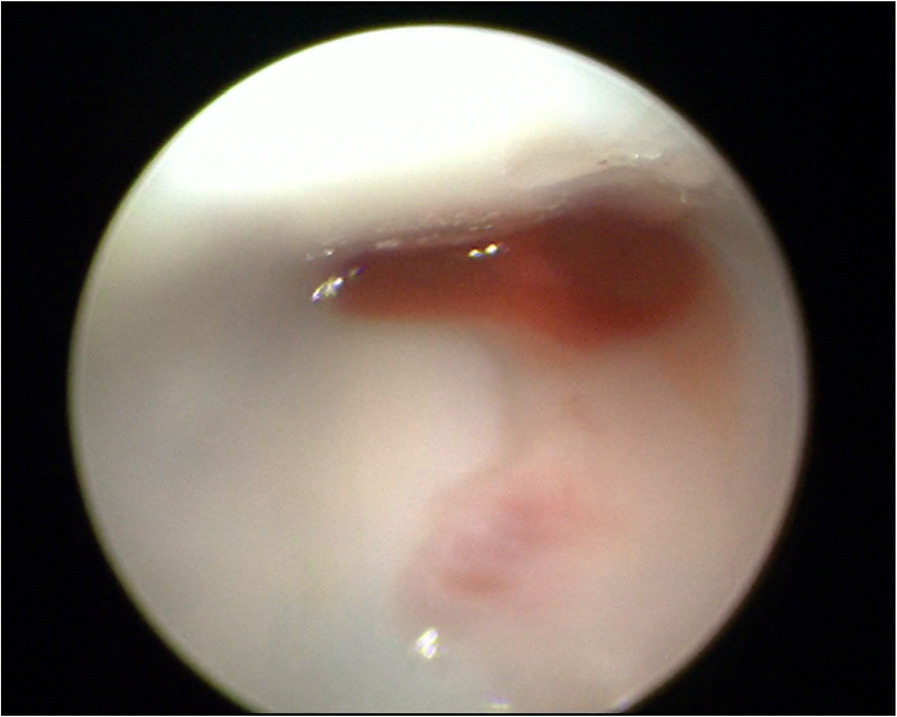
Image showing that the protective sleeve is pressed into an oblate circle in the patellofemoral joint in the absence of a PEEK trocar or metal inner protective sleeve

**Fig. 9:**
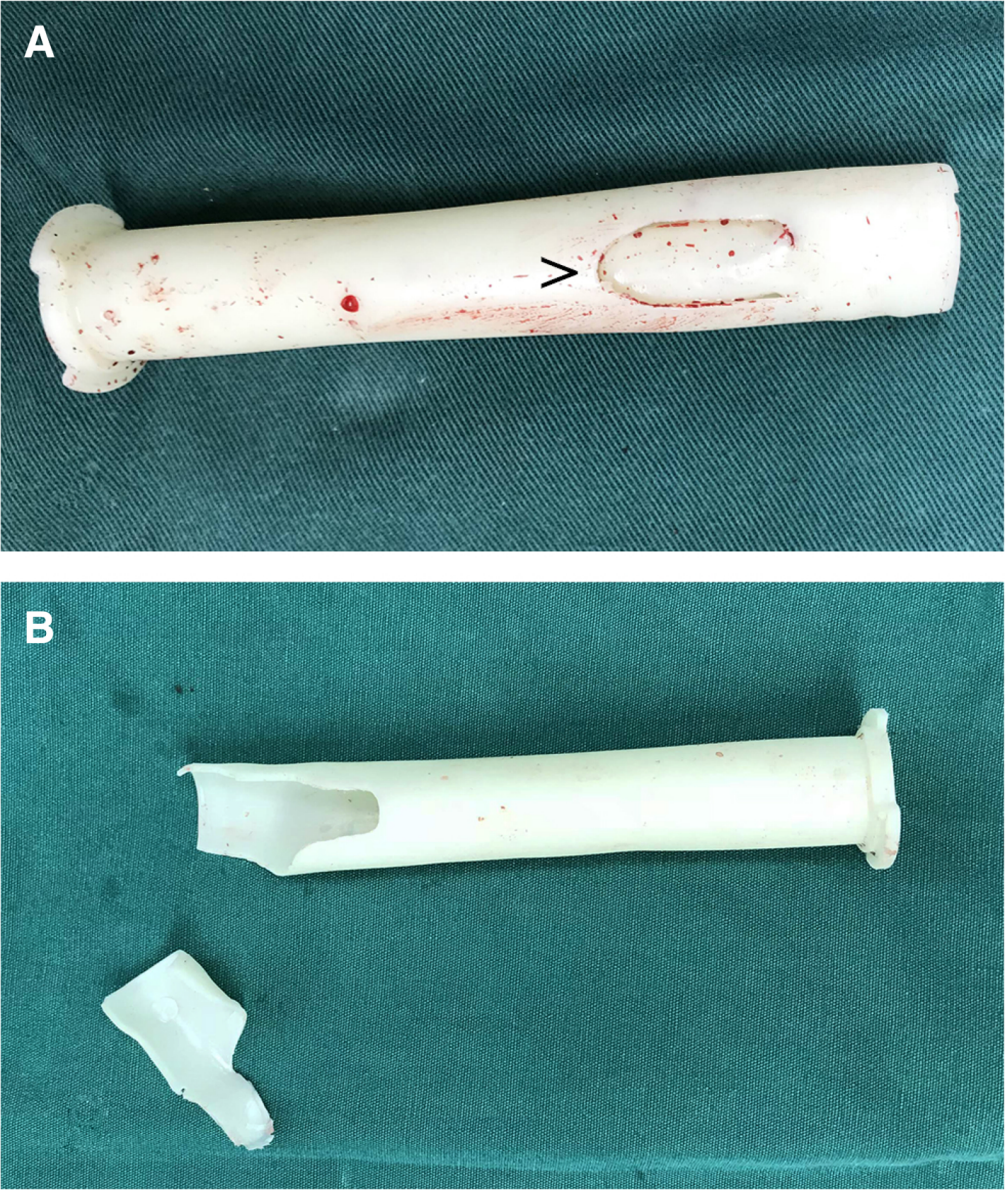
Two cases (**a** and **b**) of sleeve damage in the conventional sleeve group; “>” indicates a crack in the sleeve

In the CASS group, the patellofemoral joint cartilage of 29 patients was not damaged postoperatively (Fig. 7); only one patient experienced cartilage damage. In the conventional sleeve group, postoperative patellofemoral joint cartilage damage was found in eight patients. Comparing the cartilage damage rate between both groups revealed *P* = 0.0301; thus, the difference was statistically significant (Table 2). During arthroscopic assessment, we also measured the pressure exerted on the protective sleeve inside the patellofemoral joint in the absence of a polyetheretherketone (PEEK) trocar or metal inner protective sleeve (Fig. 8). Two of the cases of articular damage in the conventional sleeve group were caused by damage to the sleeve (Fig. 9), resulting in a change in the Outerbridge classification from a grade of “0,” preoperatively, to a grade III, postoperatively.

**Table 2:** Comparison of cartilage damage rate between 30 patients in the Chinese Aircraft-shaped Sleeve group and 30 patients in the conventional sleeve group

Group	Positive	Negative	Total	Residual rate (%)	*P* value*
Chinese Aircraft-shaped Sleeve group	1	29	30	3.33	0.0301
Conventional sleeve group	8	22	30	26.67	
Total	9	51	60	15.00	

*Chi-square test with correction for continuity in a 2 × 2 table

On arthroscopic assessment, residual debris was identified, after irrigation, in one case in the CASS group compared to five cases in the conventional sleeve group (*P* = 0.197, calculated using the chi-square test with correction for continuity in a 2 × 2 table; Table 3).

**Table 3:** Comparison of postoperative residual rate of debris between 30 patients in the Chinese Aircraft-shaped Sleeve group and 30 patients in the conventional sleeve group

Group	Positive	Negative	Total	Residual rate (%)	*P* value*
Chinese Aircraft-shaped Sleeve group	1	29	30	3.33	0.1967
Conventional sleeve group	5	25	30	16.67	
Total	6	54	60	10.00	

*Chi-square test with correction for continuity in a 2 × 2 table

## Discussion

The suprapatellar approach is initiated through the patellofemoral joint gap. As such, the relatively smaller body size of Chinese individuals, compared to Westerners, reduces the joint space available for manoeuvring and insertion of the intramedullary nail. It is to address this specific issue of size difference between two ethnic populations that we developed the CASS based on reliable anatomical parameters of the patellofemoral joint measured in a sample group of 212 individuals using MRI. MRI was selected as it effectively displays cartilage and periarticular soft tissues [6]. MRI also provides images in multiple views, which facilitates observation of the patellofemoral joint from various angles, accurately displaying the area of contact between the patella and the femoral sulcus [7]. It has been suggested that the sulcus angle is consistently smaller when measured from subchondral bone than from cartilage (5–7°). Moreover, MRI is the most suitable tool for longitudinal studies of the femoral sulcus [8]. Therefore, the MRIs of the knee joints of 212 healthy Chinese people were evaluated; measurements among women were significantly smaller than those among men (*P* < 0.05).

Although soft tissue has a certain degree of elasticity and the patella is lifted after incising the quadriceps, it is difficult to insert a protective sleeve into the narrow patellofemoral joints of Chinese surgical patients, and this is especially true of Chinese women, which increases operative time. Moreover, we observed greater pressure being exerted on the protective sleeve inside the patellofemoral joint (Fig. 8). Careless or forceful insertion of an intramedullary nail through a deformed sleeve increases the risk for iatrogenic injury. In our study, two patients in the conventional sleeve group experienced articular cartilage damage because of damaged sleeves (Fig. 9). Yasuda et al. [9] proposed an improved parapatellar approach for the narrow patellofemoral joint gap. This approach includes a 5–6-cm skin incision made on the lateral side of the patella and slitting of the lateral patellar retinaculum until the joint capsule can be opened, with a 2–0 absorbable monofilament suture used to achieve and maintain patellar eversion. Following this alternate procedure, conventional tibial reaming and IMN were performed. However, this approach to increasing the surgical workspace requires cutting the retinaculum surrounding the patella. In a cadaver study, Sandmeier et al. [10] compared the patellar tracking status of intact, incised, and reconstructed medial patellar retinacula. With the application of a lateral stress, patellar tracking changed significantly after medial retinaculum incision. After reconstruction, patellar tracking improved, but could not be restored to a completely normal state. Therefore, we believe that the suprapatellar approach to retinaculum incision for patellar subluxation requires further study.

Another difficulty that has previously been encountered during positioning is related to the use of a circular hollow sleeve to protect the patellofemoral joint in the traditional suprapatellar approach. Tajima et al. [3] designed a heart-shaped outer sleeve for the suprapatellar approach. The ventral side of the sleeve was affixed to the posterior articular surface of the patella at 130°. The dorsal side was affixed to the femoral sulcus at 140°. In our measurement of anatomical parameters, we determined that these angles are inappropriate for Chinese individuals, with a PA of 135.40 ± 6.20° and SA of 142.37 ± 5.33° when the medial and lateral cartilage surfaces of the patella and sulcus, respectively, meet in the middle. Based on these measurements, we designed an anatomically correct protective outer sleeve (aircraft wings), with a ventral face angle of 135° and the dorsal face angle of 142°.

**Fig. 10:**
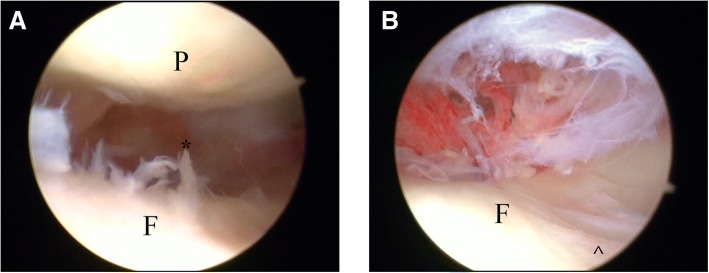
Cartilage injury observed by arthroscopy in the conventional sleeve group. **a** “*” Cartilage fragmentation and fissuring. **b** “^” Cartilage displays injury in the shape of a ski trail

Currently, some scholars are concerned that the suprapatellar approach may cause iatrogenic injuries [11–15]. In this study, cartilage damage was also observed in the conventional sleeve group (Fig. 10), and the rate was higher than that in the CASS group. It may be that the patellar gaps in persons of Chinese descent are small, leading to iatrogenic injury when sleeves or intramedullary nails are inserted. The main methods of reducing the above iatrogenic injuries include (1) using the aircraft cabin advance positioning technology; (2) retaining the protective sleeve intraoperatively; (3) preventing the knee joint from being straightened when the nail is being inserted; (4) avoiding forceful separation of the outer sleeve and the inner metal sleeve or PEEK trocar, as the outer sleeve is soft and would be deformed by pressure if it exists in isolation in a narrow patellofemoral joint; (5) stopping the reamer rotation and lifting up the drill to centre the reamer shaft in the sleeve, then moving the reamer in and out until the reamer comes out in a situation wherein the reamer is caught on the edge of the inner protection sleeve because of its sharp angle of entry into the tibia; and (6) using general anaesthesia when certain muscle relaxation is required.

The suprapatellar approach is performed through the joint cavity, and debris are generated when open reaming is performed. If debris remain in the joint cavity after the intraoperative procedures, there is a risk of heterotopic ossification or joint mice. Sanders et al. [13] reported that debris does not form new bones in synovial fluid; hence, heterotopic ossification is not a serious problem, but thorough postoperative irrigation is still necessary. In the conventional suprapatellar approach, the flow rate is limited, the direction of flow is not controllable, and blind angles are not easily irrigated. Consequently, the irrigation time tends to be long and the process may not be sufficiently thorough to avoid postoperative issues. To avoid this, it is mandatory to use a large amount of saline to perform pressurised irrigation in all directions. Currently, there are no relevant studies to indicate the amount of solution that is needed. We referred to a study by Niki et al. [16] that investigated the amount of saline needed for effective irrigation in total knee arthroplasty. Their results showed that 4000 ml of pulsed irrigation was the most efficient method of removing bone and bone cement debris. Therefore, in this study, we also used 4000 ml of rinsing solution. Our conventional sleeve group had a total irrigation time of 16.7 ± 0.9 min, which was considered a long time. It was clear that in routine surgery, manual irrigation using 4000 ml of saline would be too time-consuming and laborious. Operating the aircraft tail combined with the pulsed irrigation is much simpler. Pressure causes the fluid to flow at a certain frequency and shooting force. The debris and bacteria in the joint cavity are separated from the tissue by the oscillating action of the pulsed water flow. During irrigation, the irrigation solution is drawn out through the aspiration channel. The total irrigation time in the CASS group was 6.9 ± 0.8 min, which was greatly reduced compared to that of the conventional group. Furthermore, the more irrigation solution used, the greater the reduction of irrigation time.

This study had several limitations, including the small sample size and insufficient postoperative follow-up time. In the future, a randomised controlled study based on a larger sample should be performed. Additionally, 4 L irrigation would be too time-consuming and laborious in routine surgery. We would recommend that 1–2 L of irrigation fluid would be more suitable for efficient removal of bone debris during tibial IMN. The most appropriate amount of irrigation needs to be determined in further studies.

## Conclusion

This study demonstrated that the CASS system provides several advantages, including simplicity in the positioning of the protective sleeve, stability of the sleeve after insertion, minimal articular cartilage damage, convenient intraoperative irrigation, and shorter surgical times. This novel and practical improvement may bring about beneficial outcomes for tibial shaft fractures using the suprapatellar approach during intramedullary nailing, especially for the treatment of smaller patients with tibial shaft fractures.

## Abbreviations

CASS: Chinese Aircraft-shaped Sleeve
IMN: Intramedullary nailing
MRI: Magnetic resonance image
PA: Patellar angle
PEEK: Polyetheretherketone
SA: Sulcus angle
SD: Sulcus depth
SW: Sulcus width
TU: Thermoplastic polyurethane
